# Dissociated skeletal response to first-line immunotherapy in synchronous bone metastatic non-small cell lung cancer patients: Clinical profiles and survival benefit of denosumab adjunction

**DOI:** 10.1016/j.jbo.2026.100797

**Published:** 2026-08-17

**Authors:** E. Massy, N. Stacoffe, M. Fauvernier, M. Duruisseaux, E. Grolleau, M. Lamkhioued, M. Pérol, C. Domblides, M. Roche, A. Stuani, P. Clézardin, E. Bonnelye, J.B. Pialat, N. Girard, C. Confavreux

**Affiliations:** a Département de Rhumatologie Sud, Centre Expert des Métastases Osseuses (CEMOS), Hôpital Lyon Sud, Institut de Cancérologie des Hospices Civils de Lyon, Pierre-Bénite F-69495, France; b Centre de Recherche en Cancérologie de Lyon (CRCL) - UMR INSERM 1052 CNRS 5286, Université Claude Bernard Lyon 1, Université de Lyon, Lyon F-69100, France; c Service de Radiologie, Hôpital Lyon Sud - Centre Expert des Métastases Osseuses (CEMOS), Hôpital Lyon Sud, Institut de Cancérologie des Hospices Civils de Lyon, Pierre-Bénite F-69495, France; d UMR CNRS 5558 LBBE, Université Claude Bernard Lyon 1, Université de Lyon, Lyon F-69100, France; e Service de Pneumologie, Hôpital Louis Pradel, Institut de Cancérologie des Hospices Civils de Lyon, Bron F-69500, France; f Service de Pneumologie, Hôpital Lyon Sud, Institut de Cancérologie des Hospices Civils de Lyon, Pierre-Bénite F-69495, France; g Service de pneumologie, Hôpital de la Croix rousse, Hospices Civils de Lyon, Lyon F-6004, France; h Département de Cancérologie Médicale, Centre Léon Bérard, Lyon F-69008, France; i Service d'Oncologie, CHU de Bordeaux, F-33000 Bordeaux, France; j Plateforme Transversale de Recherche Clinique de l'Institut de Cancérologie, HCL, Pierre-Bénite F-69495, France; k LYOS INSERM UMR1033 - Université Claude Bernard Lyon 1, Université de Lyon, Lyon F-69100, France; l Canther-cancer heterogeneity, plasticity and resistance to therapies, CNRS, Inserm, CHU Lille, Institut Pasteur de Lille, UMR9020-UMR1277, Lille, France; m Institut Curie, Department of Medical Oncology, Paris, F-75000 France — Université Paris-Saclay, Université Versailles Saint-Quentin-en-Yvelines, Versailles, F-78000, France; n Service de Biostatistiques-Bioinformatique, Pôle de Santé Publique, Hôpital Lyon Sud, Pierre-Bénite F-69495, France

**Keywords:** Non-small cell lung Cancer, Bone metastases, Immune checkpoint inhibitors, Dissociated response, Denosumab

## Abstract

**Introduction:**

Patients with bone metastases (BM) from non-small cell lung cancer (NSCLC) derive less clinical benefit from immune checkpoint inhibitors (ICIs) than those without skeletal involvement. We aimed to characterize bone-specific disease control profiles under first-line ICI therapy and explore the association of denosumab with clinical outcomes.

**Methods:**

We conducted a multicenter retrospective study in BM-NSCLC patients, naïve to systemic therapy, treated with first-line ICI monotherapy, with or without denosumab. Radiological response in soft tissue and bone compartments was assessed at 6 months using iRECIST. Disease control (DCR: iCR, iPR, or iSD) and dissociated response (discordant DCR between compartments) were predefined. Denosumab impact was evaluated using logistic regression and Cox models.

**Results:**

Sixty-one patients were included (16 women [26%]; mean age 65.8 ± 9.6 years). Soft tissue DCR was 64% and bone DCR was 62% at M6. Dissociated response occurred in 21% (*n* = 13), associated with female sex, *KRAS* mutations, and elevated C-reactive protein (all *p* < 0.05). Denosumab did not alter bone DCR (73% vs. 60%; adjusted OR 0.64; 95% CI: 0.12–2.99; *p* = 0.582) but was associated with improved progression-free survival (log-rank *p* = 0.06) and reduced hazard of progression or death (adjusted HR 0.28; 95% CI: 0.06–1.33; *p* = 0.110).

**Conclusion:**

ICI-driven immune activation does not translate uniformly across compartments. Female sex, *KRAS* mutation, and systemic inflammation are potential determinants of bone-specific resistance. While denosumab did not significantly modify bone DCR, its trend toward association with improved progression-free survival and reduced risk of progression or death generates the hypothesis of an immunomodulatory effect warranting prospective evaluation.

## Highlights

• Dissociated ICI response (bone vs. soft tissue) occurs in 21% of bone-metastatic NSCLC.
• Female sex, *KRAS* mutation, and high CRP predict bone-specific ICI resistance.
• Bone and soft tissue DCR are comparable at 6 months (62% vs. 64%) with first-line ICI.
• Denosumab trends toward better PFS and 55% reduced progression/death hazard with ICI.
• RANKL blockade may act as immune sensitizer in bone niche beyond antiresorptive role.

## 1 Introduction

Bone metastases (BM) represent a frequent and severe complication of advanced non-small cell lung cancer (NSCLC), occurring in 30–40% of patients and associated with significant skeletal morbidity and reduced survival [1], [2], [3]. Once established, cancer cells disrupt bone homeostasis by hijacking osteoblast and osteoclast functions, creating a self-amplifying “vicious cycle” of skeletal destruction and tumor growth [4].

The advent of immune checkpoint inhibitors (ICIs), particularly anti-PD-1 antibodies, has transformed the management of metastatic NSCLC [5]. However, accumulating evidence suggests that patients with BM derive substantially less benefit from ICI therapy than those without bone involvement [6], [7], [8]. Retrospective analyses consistently report shorter progression-free survival (PFS) and overall survival (OS) in bone-metastatic patients, with median OS dropping from 13.4 to 5.9 months in the presence of BM [9], [10]. These observations point to a compartment-specific resistance to immunotherapy within the bone microenvironment.

Several large trials that could have addressed bone-specific ICI efficacy were confounded by methodological limitations: heterogeneous populations mixing treatment lines, prior exposure to antiresorptive agents, or absence of compartment-specific response assessment. As a result, dissociated radiological responses, where soft tissue lesions respond while bone lesions progress, or vice versa,have been repeatedly observed but never formally studied in a controlled manner [8]. Similar dissociated response patterns have been described across other tumor types with bone involvement, including renal, urothelial and prostate cancer, suggesting a pan-cancer biological phenomenon [11], [12], [13], [14], [15].

This compartment-specific resistance is thought to reflect the intrinsic immunosuppressive character of the bone microenvironment, shaped by multiple converging mechanisms: recruitment of myeloid-derived suppressor cells (MDSCs) and tumor-associated macrophages, TGF-β release during osteoclast-mediated resorption promoting Th17 over Th1 responses, and the immunomodulatory consequences of oncogenic drivers such as EGFR mutations or ALK rearrangements [16], [17], [18].

In this context, the addition of denosumab (an anti-RANKL monoclonal antibody) to ICI-based regimens has attracted interest as a strategy to simultaneously target bone destruction and restore immune competence within the bone niche. Beyond its antiresorptive role [19], denosumab may enhance ICI efficacy by disrupting RANKL-mediated immunosuppression and increasing CD8+ T cell infiltration [20], [21]. Preliminary clinical evidence supports a survival benefit from combining denosumab and ICIs in bone-metastatic NSCLC patients [22], [23], [24], though no study has yet specifically evaluated bone response rates in a homogeneous, treatment-naive cohort.

To address this gap, we conducted a multicenter retrospective study in a tightly defined population: patients with synchronous bone-metastatic NSCLC at diagnosis, naive to both systemic oncological therapy and antiresorptive agents, and treated with first-line ICI monotherapy. This rigorous methodological approach was designed to specifically isolate the true bone response to ICI therapy, out of other potential confounding treatments. The objectives were to characterize bone-specific disease control profiles, identify predictors of dissociated responses, and explore the association of denosumab with clinical outcomes in this population.

## 2 Methods

### 2.1 Study design and population

This multicenter retrospective cohort study was conducted across four French clinical centers: Lyon Sud University Hospital (Hospices Civils de Lyon), Léon Bérard Cancer Center (Lyon), Institut Curie (Paris), and Bordeaux University Hospital. Patients were enrolled consecutively between 2017 and 2022. We included adult patients with histologically confirmed NSCLC and synchronous BM at diagnosis who were treated with first-line ICI monotherapy. Patients who were minors or who had formally expressed opposition to the use of their clinical data were excluded. Patients may have additionally received antiresorptive agents (denosumab or zoledronic acid). Comparisons between patients who received denosumab and those who received no antiresorptive agent at first-line ICI initiation excluded the single patient treated with zoledronic acid, restricting this comparison to 60 patients (45 with no antiresorptive agent, 15 with denosumab). Three patients from the no-antiresorptive-agent group subsequently started denosumab during follow-up, following a bone-related clinical event; they were retained in the no-antiresorptive-agent group for this comparison, which specifically evaluates exposure at treatment initiation.The primary objective was to characterize the evolution of BM response after 6 months of therapy.

### 2.2 Radiological assessment

A standardized retrospective analysis of computed tomography (CT) scans was performed at three specific time points: baseline (before treatment initiation), month 3 (M3), and month 6 (M6). All images were centrally blind reviewed by a radiologist expert in osteo-articular imaging to ensure precise and unbiased evaluation of bone metastasis. Bone staging at baseline was performed using thoraco-abdomino-pelvic CT scan, complemented by cerebral imaging and metabolic imaging (PET-FDG) according to standard clinical practice at each center.

### 2.3 Response assessment and scoring

Tumor response was assessed in both soft tissue (extraosseous) and bone lesions using the iRECIST criteria [25]. A measurable bone lesion was defined as an osteolytic, mixed, or sclerotic lesion with a soft tissue component greater than 10 mm. Radiological response was categorized into five stages: immune complete response (iCR), immune partial response (iPR), immune stable disease (iSD), immune unconfirmed progressive disease (iUPD), and immune confirmed progressive disease (iCPD). Specifically, iCR refers to the disappearance of all target and non-target lesions, while iPR is defined as at least a 30% reduction in the sum of the diameters of target lesions compared to baseline. iSD represents a state where there is neither sufficient shrinkage to qualify for iPR nor a sufficient increase to qualify for progression. iUPD occurs upon the first identification of a 20% or greater increase in tumor burden or the appearance of new lesions, whereas iCPD requires confirmation of this progression by a follow-up scan performed at least 4 to 8 weeks later. For the primary analysis, disease control was defined as achieving iCR, iPR, or iSD hereafter referred to as the disease control rate (DCR) while non-control was defined as iUPD or iCPD. The inclusion of iSD in the DCR definition reflects the immunotherapy-specific response kinetics where prolonged stable disease represents a meaningful clinical benefit. A dissociated response was defined as a discrepancy in response status between soft tissue and bone targets at M6, encompassing both situations: response in soft tissue with progression in bone, or conversely, response in bone with progression in soft tissue.

### 2.4 Data collection and clinical variables

Baseline clinical and biological data were retrieved from medical records, including age, sex, tobacco status, body mass index (BMI), histology and Performance Status (PS). The presence of extraosseous metastases (brain, liver, lung, adrenal, or muscle) and PD-L1 expression levels (categorized as negative [<1%], low [1–49%], or high [≥50%]) were also recorded. C-reactive protein (CRP) levels and tumor mutational status (*EGFR*, *KRAS*, *ALK*, *MET, BRAF, ROS*), were documented at treatment initiation. Treatment with bone-targeted agents (denosumab, zoledronic acid), the use of corticosteroids (>10 mg prednisone equivalent at baseline) and the occurrence of immune-related adverse events (irAEs) were also recorded. For patients receiving denosumab, the total number of administration cycles and treatment duration were recorded. The occurrence of osteonecrosis of the jaw or severe hypocalcemia leading to discontinuation of the antiresorptive agent was specifically screened for among all antiresorptive-treated patients.

### 2.5 Statistical analysis

Categorical variables were expressed as counts and percentages, while quantitative variables were expressed as mean ± standard deviation (SD) or median with interquartile range (IQR). Clinical characteristics were compared between patients with parallel and dissociated responses using Fisher's exact test for categorical data and Mann-Whitney U or Student's *t*-test for continuous variables, as appropriate. The association between soft tissue and bone disease control was evaluated using Fisher's exact test to estimate Odds Ratios (OR). To assess the effect of denosumab on bone DCR and soft tissue DCR at M6, adjusted logistic regression models were performed, incorporating potential confounding factors: PD-L1 expression, brain metastases, liver metastases, age, PS, and corticosteroid use. These models were restricted to patients who received denosumab or no antiresorptive agent, excluding the single patient treated with zoledronic acid. PFS was analyzed using Kaplan-Meier curves; differences between groups (ICI alone vs. ICI + denosumab) were evaluated using the log-rank test. An adjusted Cox proportional hazards model was used to estimate the hazard of progression or death. Given the exploratory nature of this study and the small sample size, all *p*-values are reported without correction for multiple testing and should be interpreted accordingly. All statistical analyses were performed with a significance level set at *p* < 0.05.

### 2.6 Ethical approval

The study was conducted in accordance with the ethical principles of the Declaration of Helsinki. Institutional Review Board approval was obtained on August 29, 2022 (No. 22_955). Data collection and processing were performed in compliance with the General Data Protection Regulation (GDPR) and the requirements of the French Data Protection Authority (CNIL, MR004). All participants received detailed information regarding the retrospective nature of the study. Only patients who did not oppose re-use of their clinical and radiological data were included.

## 3 Results

### 3.1 Population characteristics

The study included 61 patients diagnosed with synchronous bone-metastatic NSCLC (Table 1). The mean age was 65.8 ± 9.6 years; 45 patients (74%) were male and 16 (26%) were female. At ICI treatment initiation, 86% of patients (*n* = 51) had a PS of 0 or 1. Regarding histology, 80% (*n* = 49) had adenocarcinoma and 16% (*n* = 10) had squamous cell carcinoma. PD-L1 expression was ≥50% in 92% of cases (*n* = 56), 1–49% in 4 patients (7%), and < 1% in 1 patient (2%). The molecular profile revealed KRAS mutations in 33% of patients (*n* = 20), EGFR mutations in 2% (*n* = 1), and ALK rearrangements in 2% (*n* = 1). At the time of diagnosis, bone involvement comprised a single lesion in 47% of cases, two lesions in 37%, and more than two lesions in 16%. Other initial metastatic sites included the lung (30%), brain (22%), liver (20%), and adrenal glands (18%). The mean baseline C-reactive protein (CRP) level was 48.6 ± 42.4 mg/L.

**Table 1:** Baseline characteristics.

	Overall (*n* = 61)	Men (*n* = 45)	Women (*n* = 16)
**Center**			
Hospices Civils de Lyon (%)	20 (33)	16	4
Institut Curie (%)	23 (38	16	7
Centre Leon Berard (%)	14 (23)	9	5
Bordeaux (%)	4 (6)	4	0
**Patients** Women (%)	16 (27)		
Age (years, mean ± SD)	65.8 ± 9.6	66.6 ± 9.8	63.7 ± 8.9
BMI (kg/m^2^, mean ± SD)	24.4 ± 4.4	24.6 ± 4.3	23.9 ± 4.9
Tobacco use	53 (87)	40 (89)	13 (81)
PS 0–1, n (%)	51 (86)	37 (84)	14 (93)
**Histology**			
Adenocarcinoma	49 (80)	34 (76)	15 (94)
Squamous cell carcinoma	10 (16)	9 (20)	1 (6)
Other	2 (3)	2 (4)	0 (0)
**PD-L1 expression**			
PD-L1 ≥ 50%, n (%)	56 (92)	43 (96)	13 (81)
PD-L1 1–49%, n (%)	4 (7)	2 (4)	2 (12)
PD-L1 < 1%, n (%)	1 (2)	1 (2)	0 (0)
**Molecular profile**			
*KRAS* mutation	20 (33)	13 (29)	7 (44)
*EGFR* mutation	1 (2)	1 (2)	0 (0)
*ALK* rearrangement	1 (2)	0 (0)	1 (6)
Other	30 (49)	26 (58)	4 (25)
**Number of bone lesions**	1.8 ± 1.3	1.9 ± 1.5	1.5 ± 0.6
1	28 (47)	19 (42)	9 (56)
2	22 (37)	16 (36)	6 (38)
>2	11 (16)	9 (20)	2 (13)
CRP (mg/L, mean)	48.6 ± 42.4	50.5 ± 42.2	46.7 ± 44.4
>10 mg prednisone equivalent (%)	13 (21)	10 (22)	3 (19)
**Initial metastatic status**			
Lung	18 (30)	16 (36)	2 (12)
Brain	13 (22)	5 (11)	8 (50)
Liver	12 (20)	8 (18)	4 (25)
Adrenal gland	11 (18)	9 (20)	2 (12)
Muscle	2 (3)	2 (4)	0 (0)
**Antiresorptive agents**			
Denosumab (%)	15 (25)	12 (27)	3 (19)
Zoledronic acid (%)	1 (1)	1 (2)	0 (0)
None (%)	45 (74)	32 (71)	13 (81)
IrAEs (%)	19 (31)	12 (27)	7 (44)

Data are expressed as mean ± standard deviation or count (percentage).

BMI: Body Mass Index, CRP: C-Reactive Protein, IrAEs: immune related adverse event, PS: personal status.

**Fig. 1:**
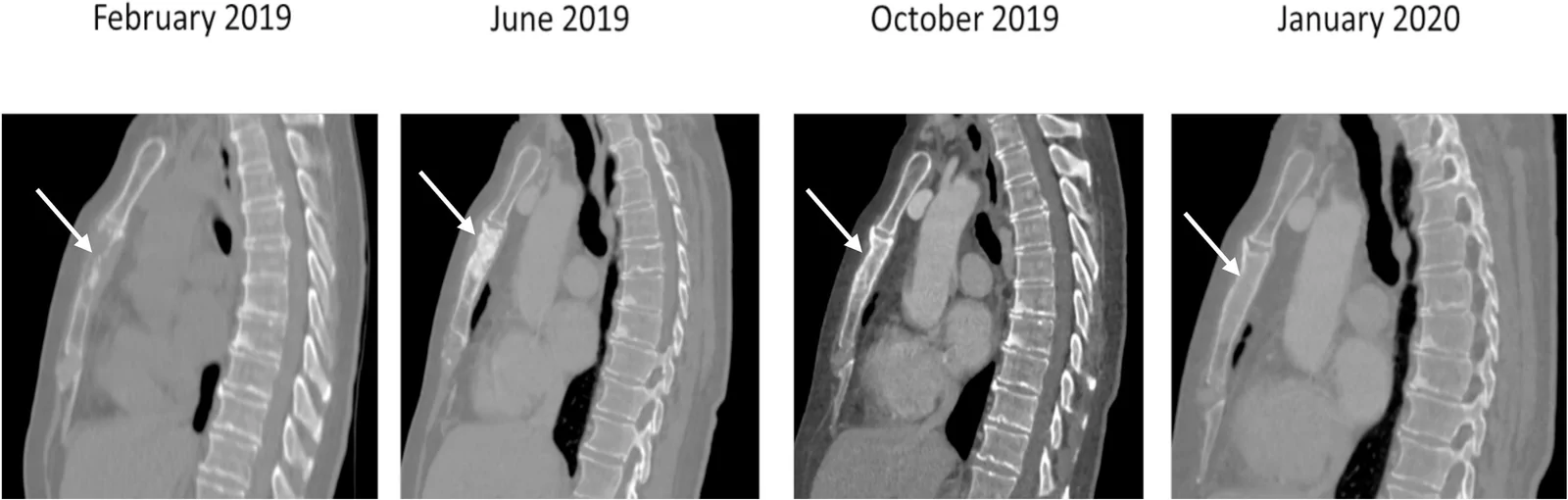
Tumor response of sternal bone metastases under immunotherapy and denosumab: serial sagittal CT scans from February 2019 to January 2020.

Compared to men, women in this cohort presented a significantly higher prevalence of brain metastases (50% vs. 11%), a greater frequency of *KRAS* mutations (44% vs. 29%), and a higher proportion of adenocarcinoma (94% vs. 76%), while all other baseline characteristics remained broadly similar between the two groups. Within this cohort, 25% of patients (*n* = 15) received denosumab in combination with first-line ICI monotherapy, and one patient received zoledronic acid (Table 1). The therapeutic potential of the ICI–denosumab combination is exemplified in Fig. 1, which shows the progressive osteoblastic response of a lytic sternal metastasis transitioning to a sclerotic pattern under combined treatment.

### 3.2 Compartment-specific disease control and dissociated responses

**Fig. 2:**
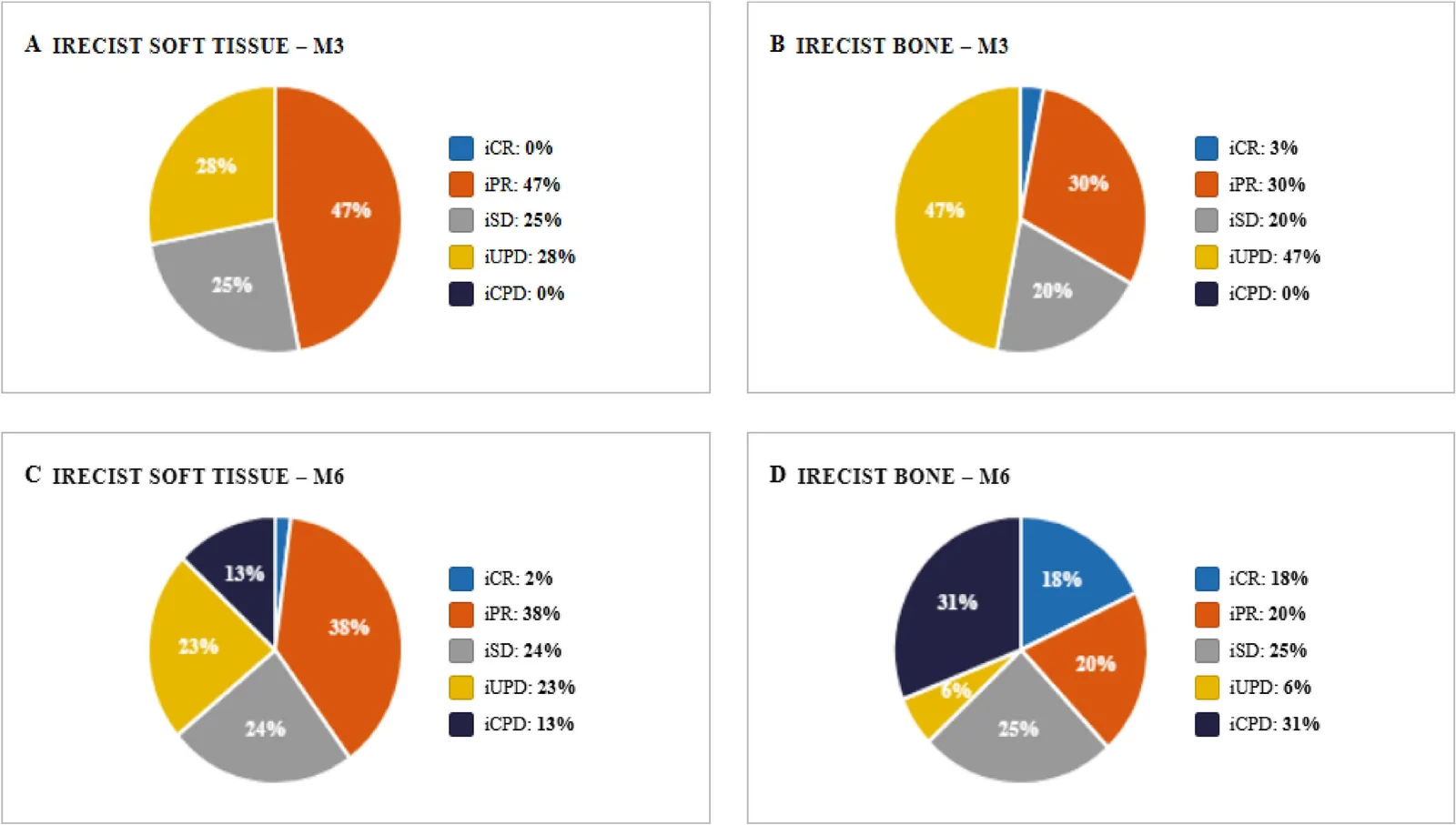
Compartment-specific iRECIST response patterns in ICI-treated NSCLC patients with bone metastases. Distribution of iRECIST responses in soft tissue (A, C) and bone (B, D) compartments at 3 months (A, B) and 6 months (C, D). iCR: Complete Response, iPR: Partial Response, iSD: Stable Disease, iUPD: Unconfirmed Progressive Disease, iCPD:Confirmed Progressive Disease, ICI: immune checkpoint inhibitor, NSCLC: non-small cell lung cancer.

At the 6 month radiological assessment, the overall soft tissue DCR was 63.9% (39/61) and the bone DCR was 62.3% (38/61) (Fig. 2). A significant association was observed between soft tissue and bone disease control (*p* = 0.0025; OR = 5.84, 95% CI: 1.70–22.09): patients achieving disease control in soft tissue had nearly 6-fold higher odds of also achieving bone disease control. Of note, the bone DCR improved from 53% (32/61) at M3 to 62% at M6, whereas the soft tissue DCR decreased slightly from 71% (43/61) at M3 to 64% at M6.

A dissociated response occurred in 21% of patients (*n* = 13), distributed in roughly equal proportions in both directions: 8 patients (62%) exhibited soft tissue disease control with bone non-control, and 5 (38%) exhibited bone disease control with soft tissue non-control. Comparative analysis between patients with parallel responses (*n* = 48) and those with dissociated responses (*n* = 13) is presented in Table 2. The proportion of women was significantly higher in the dissociated group (46% vs. 21%; *p* < 0.05). KRAS mutations were present in 62% of patients with a dissociated response versus 25% in those with parallel responses (*p* < 0.05). The mean baseline CRP was significantly higher in the dissociated group (62.2 ± 30.9 mg/L) than in the parallel group (46.3 ± 44.6 mg/L; *p* < 0.05). Furthermore, the absence of lung metastases at diagnosis was significantly associated with a dissociated response pattern (8% vs. 35%; *p* < 0.05). Conversely, no differences were observed for histology, age, BMI, smoking status, PS, and number of bone lesions.

**Table 2:** Baseline characteristics of patients according to response pattern to immunotherapy.

	Overall	Parralel response	Dissociated response	p
Number of patients (n)	61	48	13	
Women (%)	16 (27)	10 (21)	6 (46)	<0.05
Age (years, mean, SD)	65.8 ± 9.6	66.2 ± 9.2	64.5 ± 11.1	Ns
BMI (kg/m^2^, mean, SD)	24.4 ± 4.4	24.1 ± 4.1	25.6 ± 5.3	Ns
Tobacco use	53 (87)	41 (85)	12 (92)	Ns
PS 0–1, n (%)	53 (87)	40 (87)	11 (85%)	Ns
Histology				
Adenocarcinoma	49 (80)	37 (77)	12 (92)	Ns
Squamous cell carcinoma	10 (16)	9 (19)	1 (8)	Ns
Other	2 (3)	2 (4)	0 (0)	Ns
PD-L1 ≥ 50%, n (%)	56 (92)	46 (96)	11 (85)	Ns
*KRAS* mutation (n, %)	20 (33)	12 (25)	8 (62)	<0.05
Number of bone lesions				
>1	33 (54)	27 (56)	6 (46)	Ns
CRP (mg/L)	48.6 ± 42.4	46.3 ± 44.6	62.2 ± 30.9	<0.05
Initial metastatic status				
Lung	18 (30)	17 (35)	1 (8)	<0.05
Brain	13 (22)	10 (21)	3 (23)	Ns
Liver	12 (20)	10 (21)	2 (15)	Ns
Adrenal gland	11 (18)	9 (19)	2 (15)	Ns
Muscle	2 (3)	2 (4)	0	Ns
Denosumab		14 (29)	1 (8)	Ns

Data are expressed as mean ± standard deviation or count (percentage).

BMI: Body Mass Index, CRP: C-Reactive Protein,Ns: non-significant, PS: performance status.

### 3.3 Association of denosumab with treatment outcomes

The impact of denosumab was assessed across response compartments and survival outcomes (Table 3). At M3, soft tissue DCR rates were comparable between the ICI + denosumab and no antiresorptive agent groups (73% vs. 71%), as were bone DCR rates (60% vs. 51%). At M6, the soft tissue DCR was 80% in the denosumab group versus 60% in the no antiresorptive agent group (adjusted OR 2.68; 95% CI: 0.45–19.68; *p* = 0.291), and the bone DCR was 73% versus 60% (adjusted OR 0.64; 95% CI: 0.12–2.99; *p* = 0.582). Neither difference reached statistical significance, and these results should be interpreted as exploratory given the small size of the denosumab subgroup (*n* = 15) and the correspondingly wide confidence intervals.

**Table 3:** Baseline characteristics and treatment outcomes of NSCLC patients with bone metastases treated with ICI monotherapy (without anti resorptive agent excluding the only patient treated with zoledronic acid) versus ICI combined with denosumab.

	Overall	ICI monotherapy	ICI + denosumab
Number of patients (n)	60	45 (75%)	15 (25%)
Center			
Hospices Civils de Lyon	20 (33)	11 (24)	8 (53)
Institut Curie	23 (38)	19 (42)	4 (27)
Centre Leon Berard	14 (23)	13 (29)	1 (7)
Bordeaux	4 (7)	2 (4)	2 (13)
**Characteristics**			
Women (%)	16 (27)	10 (21)	6 (46)
Age (years, mean, SD)	66.0 ± 9.5	66.1 ± 8.4	65.5 ± 12.8
BMI (kg/m^2^, mean, SD)	24.5 ± 4.4	24.7 ± 4.6	23.8 ± 3.9
Tobacco use	52 (87)	41 (91)	11 (73)
PS 0–1, n (%)	50 (83)	38 (84)	12 (80)
Number of bone lesions			
1	28 (47)	25 (56)	3 (20)
2	21 (35)	15 (33)	6 (40)
>2	11 (17)	5 (11)	6 (40)
CRP (mg/L, mean)	49.9 ± 42.7	41.2 ± 34.6	75.8 ± 54.7
Bone resorptive agent			
Denosumab (%)	15 (25)	0	15 (100)
Zoledronic acid (%)	0	0	0
IrAEs (%)	19 (31)	12 (27)	7 (47)
10 mg prednisone equivalent (%)	13 (21)	10 (22)	3 (20)
Initial metastatic status			
Lung	17 (28)	16 (36)	1 (8)
Brain	13 (22)	10 (22)	3 (23)
Liver	11 (18)	9 (20)	2 (15)
Adrenal gland	11 (18)	9 (20)	2 (15)
Muscle	2 (3)	1 (2)	1 (7)
DCR response			
M3 soft tissue M3	43 (71)	32 (71)	11 (73)
M6 soft tissue	39 (64)	27 (60)	12 (80)
M3 bone	32 (53)	23 (51)	9 (60)
M6 bone	38 (62)	27 (60)	11 (73)
Dissociated response	13 (21)	12 (27)	1 (7)

Data are expressed as mean ± standard deviation or count (percentage).

**Fig. 3:**
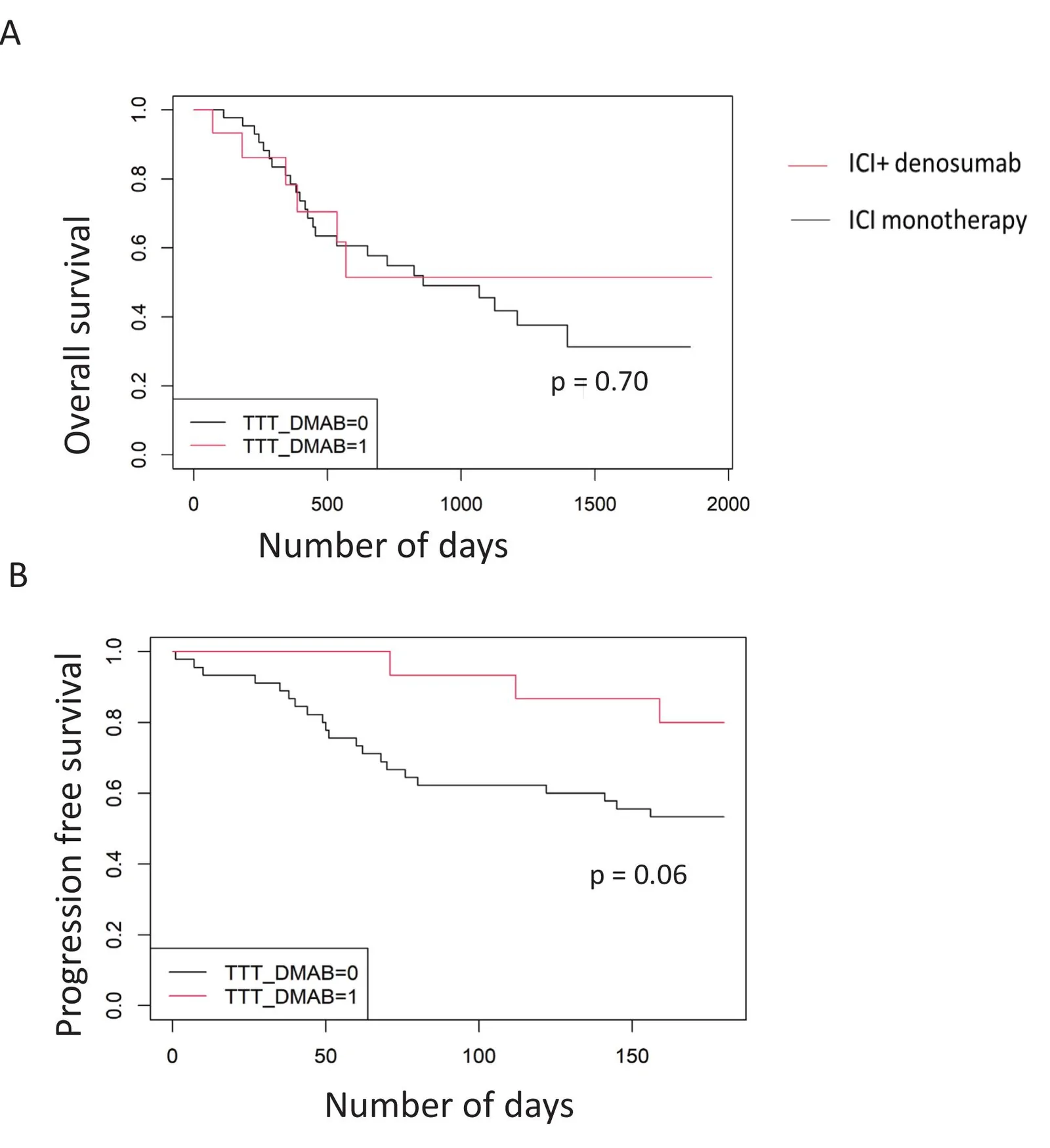
Impact of denosumab combination on survival outcomes in ICI-treated NSCLC patients with bone metastases. Kaplan-Meier curves of overall survival (A) and progression-free survival (B) according to denosumab use.

BMI: Body Mass Index, CRP: C-Reactive Protein, DCR: Disease Control Rate, ICI: immune check point inhibitor, IrAEs: immune related adverse event, PS: performance status,

Dissociated responses were less frequent in the denosumab group (7%, *n* = 1) than in the ICI monotherapy group (27%, *n* = 12). Regarding survival outcomes, no significant difference was observed in overall survival at 6 months (log-rank *p* = 0.70) (Fig. 3A). However, an unadjusted analysis of progression-free survival (PFS) showed a trend toward improved outcomes in the combination group (log-rank *p* = 0.06) (Fig. 3B). According to the adjusted Cox proportional hazards model, the addition of denosumab was associated with a 72% reduction in the hazard of progression or death (adjusted HR 0.28; 95% CI: 0.06–1.33; *p* = 0.110). These results, while not reaching conventional significance thresholds, are hypothesis-generating and consistent with an immunomodulatory effect of denosumab beyond its established antiresorptive role.

## 4 Discussion

This multicenter retrospective study analyzed bone-specific response patterns and the impact of denosumab addition in a tightly controlled population of patients with synchronous bone-metastatic NSCLC, naive to both systemic oncological therapy and antiresorptive agents, receiving first-line ICI monotherapy. This methodological stringency was essential to isolate the true immunological response within the bone compartment, free from the confounding effects of prior treatments.

Our main findings are threefold. First, despite a significant association between soft tissue and bone responses at 6 months, approximately one in five patients exhibited a dissociated response, with divergent trajectories between compartments occurring in roughly equal proportions in both directions. Second, our exploratory findings suggest that female sex, *KRAS* mutation status, and high baseline CRP levels are associated with dissociated response profiles, signaling possible host and tumor-driven mechanisms of site-specific immunotherapy resistance. Third, while denosumab did not significantly improve the radiological bone response rate, its addition was associated with a numerical trend toward a reduced risk of progression or death; though not reaching statistical significance, this observation may suggest potential immunomodulatory effects extending beyond its antiresorptive role.

Taken together, these results underscore the clinical complexity of bone metastatic disease under ICI therapy, where radiological response and survival benefit can be dissociated. They also support the integration of denosumab into first-line ICI regimens for bone-metastatic NSCLC patients, not solely for skeletal protection, but as a potential immune sensitizer within the bone niche.

The 21% rate of dissociated responses confirm that systemic immune activation by ICIs does not translate uniformly across anatomical sites. This is consistent with the findings of Xu et al. [26], who demonstrated that progression under ICI in NSCLC is frequently organ-specific, with 64% of bone progressions classified as oligo-progression (suggesting localized therapeutic resistance within the bone compartment) despite disease control elsewhere. The bone marrow constitutes a unique immune niche, naturally enriched with myeloid-derived suppressor cells (MDSCs, representing up to 30% of its cellular content) and depleted of mature cytotoxic T lymphocytes [16], [20]. Recent literature has further highlighted the role of immature neutrophils, rapidly recruited to bone metastases, which directly inhibit CD8+ T cell activation [27]. The bone metastatic niche is often described as “immunologically cold” [20], and cancer cells actively reinforce this state by disrupting bone homeostasis and creating a “vicious cycle” of bone resorption that releases factors suppressing anti-tumor immunity [4]. Beyond cellular infiltration, physical degradation of the bone matrix releases TGF-β, which skews T-cell differentiation toward the pro-tumoral Th17 lineage at the expense of the protective Th1 response [4], [17]. Additionally, osteopontin produced by osteoclasts during resorption has been shown to remotely impair CD8+ T cell recruitment even at extraosseous sites, contributing to a state of systemic immunotherapy resistance in patients with high bone tumor burden [28].

To date, specific predictors of dissociated responses between bone and soft tissue compartments remain poorly characterized. Our exploratory findings point to three potential determinants: female sex, *KRAS* mutation, and elevated baseline CRP. Women may exhibit distinct compartment-specific therapeutic responses due to the complex interplay between sex hormones and the immune microenvironment. In women, dissociated responses may be further driven by the heterogeneous expression of the androgen receptor (AR) across different metastatic sites: localized AR signaling can epigenetically reprogram CD8+ T cells toward an exhausted phenotype and promote M2-like macrophage polarization, thereby creating “immune sanctuaries” in specific nodules that resist systemic therapy while others remain controlled [29]. *KRAS* mutations act as potent drivers of spatial immune heterogeneity, with their clinical behavior heavily dictated by co-occurring genomic alterations and the inflammatory state of the tumor microenvironment [30]. Moreover, *KRAS* mutations are known to modulate the immune landscape in NSCLC, potentially creating organ-specific patterns of immune resistance [6]. Finally, elevated CRP likely reflects a systemic inflammatory state associated with high bone tumor burden and an MDSCs-enriched microenvironment, both of which have been linked to ICI resistance [31], [32]. Beyond these clinical predictors, the TRACERx study provides a compelling biological framework: metastatic lineages frequently undergo saltatory evolution and develop distinct immune evasion mechanisms compared to the primary tumor, offering a clear mechanistic basis for the compartment-specific responses observed under the selective pressure of systemic therapy [33]. We also evaluated PD-L1 expression, as it remains the primary determinant of response and the standard for ICI approval in metastatic lung cancer [34]. However, in our cohort, PD-L1 expression exceeded 50% in nearly all patients, and we did not observe a significant role for this biomarker in the occurrence of dissociated response patterns.

One of the most striking findings of this study is the dissociation between the radiological and clinical impacts of denosumab. While its addition did not significantly increase the rate of radiological bone DCR at M6, combination therapy was associated with a clear trend toward improved PFS and a reduction in mortality risk. This suggests that denosumab may exert immunomodulatory properties not necessarily captured by conventional radiological criteria at 6 months, and that its survival benefit operates through mechanisms extending beyond its established antiresorptive role [19]. The mechanistic basis for this benefit likely resides in the RANK/RANKL axis: RANKL is not only essential for osteoclastogenesis but is also expressed by regulatory T cells (Tregs), where it promotes an immunosuppressive phenotype within the tumor microenvironment [21], [35]. By blocking this pathway, denosumab may restore immune competence within the bone niche without necessarily translating into immediate radiological changes detectable at 6 months. Our results align with broader clinical evidence suggesting that NSCLC patients receiving combined ICI and denosumab therapy achieve better survival outcomes than those on ICI alone [22], [23], [36], including data from the IMMUCARE cohort [24]. These findings are further supported by preclinical data [21]. Notably, denosumab has been reported to outperform bisphosphonates in enhancing survival in patients with high bone tumor burden treated with ICIs [37]. Several prospective randomized controlled trials are currently ongoing to formally confirm this synergy between denosumab and ICI in lung cancer and other tumor types [20], [38].

The timing of denosumab initiation relative to ICI may also be clinically relevant. Current evidence suggests that initiating ICI therapy prior to or concurrently with denosumab may be the most effective strategy [24]. Preclinical models indicate that prior ICI exposure enhances lymphocyte infiltration before the bone microenvironment is significantly altered by the cessation of bone resorption [21]. Furthermore, ICI treatment has been shown to upregulate RANKL expression on activated CD8+ T cells [21]. This induction creates a primed environment where subsequent denosumab administration can specifically target this T-cell-derived RANKL, offering a dual therapeutic benefit: direct inhibition of osteoclastogenesis and potentiation of the anti-tumor immune response.

The high rate of dissociated responses highlights the need for a tailored monitoring strategy. Standard radiological criteria may not fully capture the complexity of the immune response in the bone. In our study, the use of iRECIST allowed for a more nuanced evaluation of stabilized disease and pseudo-progression. Clinicians should maintain high vigilance when soft tissue lesions respond but bone lesions appear stable or progress, as this profile may signal the need for early intensification with bone-targeted agents.

The primary strength of this study lies in its methodological homogeneity: a consecutive cohort of treatment-naive patients receiving first-line ICI monotherapy, with centralized blinded radiological review of all bone assessments. Several limitations must be acknowledged. The cohort size is relatively small, particularly the denosumab subgroup (*n* = 15), which substantially limits statistical power; all denosumab-related results should be considered hypothesis-generating only. The decision to combine an antiresorptive agent with first-line ICI therapy was left to individual prescriber judgment rather than a center-specific or protocol-driven algorithm, with no single center accounting for the majority of denosumab prescriptions. The retrospective design nonetheless introduces potential selection bias, as patients receiving denosumab had a higher baseline bone tumor burden (median 2 vs 1 bone lesions) and higher CRP levels than those who received no antiresorptive agent, which may confound the comparison despite multivariable adjustment. Osteonecrosis of the jaw and severe hypocalcemia leading to treatment discontinuation were specifically sought among antiresorptive-treated patients and were not identified in this cohort; however, dental and biological monitoring were not systematically protocolized during the inclusion period, and this finding should be interpreted with corresponding caution. Finally, the absence of longitudinal biological data prevents direct mechanistic correlation with the observed clinical trends.

## 5 Conclusion

Patients with BM-NSCLC treated with first-line ICIs face a complex response landscape where dissociated responses occur in a significant proportion of cases. Profiles involving female sex, *KRAS* mutations, and high baseline inflammation are particularly prone to this phenomenon. While denosumab does not appear to significantly alter the immediate radiological appearance of bone lesions, its association with improved progression free survival and reduced mortality risk suggests a potent additional immunomodulatory effect when combined with ICIs. These findings support the integration of denosumab in the management of this population and emphasize the need for larger prospective trials to optimize combined therapeutic strategies, specifically regarding sequencing and biomarker-driven decision-making.

## CRediT authorship contribution statement

**E. Massy:** Writing – review & editing, Writing – original draft, Visualization, Validation, Project administration, Methodology, Investigation, Funding acquisition, Formal analysis, Data curation, Conceptualization. **N. Stacoffe:** Writing – review & editing, Validation, Investigation. **M. Fauvernier:** Methodology, Formal analysis. **M. Duruisseaux:** Writing – review & editing, Validation. **E. Grolleau:** Writing – review & editing, Validation. **M. Lamkhioued:** Writing – review & editing, Validation. **M. Pérol:** Writing – review & editing, Validation. **C. Domblides:** Writing – review & editing, Validation. **M. Roche:** Writing – review & editing, Validation, Investigation. **A. Stuani:** Writing – review & editing, Validation, Investigation. **P. Clézardin:** Writing – review & editing, Validation. **E. Bonnelye:** Writing – review & editing, Validation. **J.B. Pialat:** Writing – review & editing, Validation. **N. Girard:** Writing – review & editing, Validation, Conceptualization. **C. Confavreux:** Writing – review & editing, Validation, Conceptualization.

## Funding

This work was supported by the Jeunes Chercheurs grant from the Hospices Civils de Lyon (2021).

## Declaration of competing interest

The authors declare the following financial interests/personal relationships which may be considered as potential competing interests: The following authors NS, MF, MD, EG, ML, CD, MR, AS, PC, EB, MP, and JBP declare that they have no known competing financial interests or personal relationships that could have appeared to influence the work reported in this paper. The authors declare the following conflicts of interests: Research grants: CC (Amgen Research Grant, Innovation research Prize, MSD avenir), EM (Dreamer Novartis Grant). Conferences: CC (Abbvie, Astra Zeneca, Celltrion, Fresenius, Sandoz, UCB, Sandoz), EM (Abbvie, Astra Zeneca, BMS,Galapagos, UCB). NG: Research grants/support to institution from Abbvie, Amgen, AstraZeneca, BeOne, Boehringer Ingelheim, Bayer, BioNTech, Bristol Myers Squibb, Daiichi-Sankyo, Gilead, J&J, Lilly, Sivan, Tahio; Consultancy: Abbvie, Accord, Amgen, AstraZeneca, BeOne, Bristol Myers Squibb, Daiichi-Sankyo, Ipsen, J&J, Lilly, Merck Sharp & Dohme, Natera, Pfizer, Pharmamar, Pierre Fabre, Regeneron, Summit; Speaker: Abbvie, Amgen, AstraZeneca, BeOne, Bristol Myers Squibb, Daiichi-Sankyo, J&J, Merck Sharp & Dohme, Novocure. Hospitality: Bristol Myers Squibb, J&J, Pierre-Fabre; Employement (family member) : AstraZeneca.
